# Identification of the Significant Genes Regulated by Estrogen Receptor in Estrogen Receptor-Positive Breast Cancer and Their Expression Pattern Changes When Tamoxifen or Fulvestrant Resistance Occurs

**DOI:** 10.3389/fgene.2020.538734

**Published:** 2020-09-29

**Authors:** Ran Cheng, Liqiang Qi, Xiangyi Kong, Zhongzhao Wang, Yi Fang, Jing Wang

**Affiliations:** Department of Breast Surgical Oncology, National Cancer Center/National Clinical Research Center for Cancer/Cancer Hospital, Chinese Academy of Medical Sciences and Peking Union Medical College, Beijing, China

**Keywords:** bioinformatics analysis, microarray, differentially expressed genes, ER-positive breast cancer, tamoxifen or fulvestrant resistance

## Abstract

Breast cancer is the most frequent malignant tumor in women, and the estrogen receptor (ER) plays a vital role in the vast majority of breast cancers. The purpose of the present study was to identify the significant genes regulated by ER in ER-positive breast cancer and to explore their expression pattern changes when tamoxifen or fulvestrant resistance occurs. For this purpose, the gene expression profiles GSE11324, GSE27473, and GSE5840 from the Gene Expression Omnibus database were used, which contain gene expression data from MCF7 cells treated with estrogen, MCF7 cells with silencing of ER, and tamoxifen- and fulvestrant-resistant MCF7 cells treated with estrogen (17β-estradiol), respectively. Differentially expressed genes (DEGs) between the treatment group and negative control were identified and subjected to pathway enrichment and protein–protein interaction (PPI) analyses. There were 230 DEGs in common among the three datasets, including 160 genes positively regulated by ER and 70 genes negatively regulated by ER. DEGs mainly showed enrichment for pathways in cancer, progesterone-mediated oocyte maturation, RNA transport, glycerophospholipid metabolism, oocyte meiosis, platelet activation, and so on. PPI network and modular analysis selected three significant clusters containing 19 genes. A total of 44 genes were involved in Kyoto Encyclopedia of Gene and Genome pathway results or PPI modular analysis, and 16 of them were found to correlate with relapse-free survival in patients with ER^+^/human epidermal growth factor receptor 2-negative breast cancer who had undergone endocrine therapies only. Some of the genes’ expression patterns were different among wild-type, tamoxifen-resistant, and fulvestrant-resistant MCF7 cells such as *DDX18*, *ANAPC7*, *MAD2L1*, *RSL1D1*, and *CALCR*, etc., indicating different resistance mechanisms and potential prognostic markers or therapeutic targets for fulvestrant- or tamoxifen-resistant breast cancer.

## Introduction

Breast cancer is the most frequently diagnosed cancer and the leading cause of cancer-related death in women worldwide (DeSantis et al., 2019). Based on current clinical findings and basic medical research, estrogen receptor (ER) exists in the majority of breast cancers and has a profound influence on the occurrence and development of breast cancer (Early Breast Cancer Trialists’ Collaborative Group et al., 2011). Endocrine therapies, such as tamoxifen, fulvestrant, and aromatase inhibitors, which target estrogen or ER, are useful in breast cancer treatment (Jelovac et al., 2005; Jordan and Brodie, 2007). However, nearly half of ER-positive breast cancer patients will eventually fail one or more of these endocrine interventions; the incidence of endocrine resistance is relatively high (Clarke et al., 2015). Therefore, more reliable endocrine therapies with efficacy- and drug resistance-related biomarkers should be explored. These biomarkers could not only be used to convey prognostic information, but may also offer breakthroughs in overcoming endocrine resistance.

Gene chips, which have been used for more than a decade, are a quick and reliable technique for exploring differentially expressed genes (DEGs) (Vogelstein et al., 2013), and large quantities of chip data have been produced and stored in public databases. These databases represent a valuable achievement that can be retrospectively analyzed based on new perspectives and approaches. Several bioinformatics studies on malignant tumors have been published in recent years, which provide new methods to uncover the underlying mechanisms of the development and progression of different types of cancers (Feng et al., 2019).

In the present study, we retrieved the gene expression profiles GSE11324, GSE27473, and GSE5840 from the Gene Expression Omnibus (GEO) as the primary research datasets. These files contain gene expression data from MCF7 cells (ER-positive breast cancer cell line) treated with estrogen, MCF7 cells with silencing of the ER, and tamoxifen- and fulvestrant-resistant MCF7 cells treated with estrogen (17β-estradiol). These datasets were utilized to identify the DEGs between the treatment group and negative control, followed by gene ontology (GO), pathway enrichment, and protein–protein interaction (PPI) analyses. Next, the interested DEGs were interrogated for prognostic information associated with the relapse-free survival (RFS) of patients with ER-positive and human epidermal growth factor receptor 2 (HER2)-negative breast cancer who had undergone endocrine therapies only, which can be the best to reflect the effect of endocrine therapies and whether patients develop endocrine resistance. Finally, we analyzed these RFS-related genes in GSE5840 gene expression profiles, which contain the differential expression gene information of tamoxifen- and fulvestrant-resistant MCF7 cells treated with estrogen. We wanted to find out significant genes regulated by ER in ER-positive breast cancer and explore their expression pattern changes when tamoxifen or fulvestrant resistance occurs. These genes may reflect the functional status of ER in endocrine-resistant breast cancer cells and promise therapeutic targets for tamoxifen or fulvestrant resistance breast cancer.

## Materials and Methods

### Microarray Data

The gene expression profiles used in this study were obtained from the NCBI-GEO, a free public data repository of microarray and other genomic data. Three ER-positive breast cancer cell line gene expression profiles, GSE11324, GSE27473, and GSE5840, were chosen as the primary research datasets. GSE11324 contains gene expression data from MCF7 cells that were stimulated with estrogen for 0, 3, 6, or 12 h. All experiments were performed in triplicate (Carroll et al., 2006). We compared the gene expression data between 0 and 3, 0 and 6, and 0 and 12 h, respectively. There are nearly consistent trends in genetic change among the three comparison groups. So we chose the 0- and 6-h time-points to compare the gene expression data and conducted the follow-up studies. GSE27473 contains gene expression data of MCF7 parental cells and MCF7 cells silencing of the ER by shRNA. These experiments were also performed in triplicate (Al Saleh et al., 2011). GSE5840 compared the gene expression patterns of 17β-estradiol-responsive genes in wild-type MCF7 cells, tamoxifen-resistant MCF7 cells, and fulvestrant-resistant MCF7 cells (the cells were treated with 17β-estradiol for 4 h). Four replicate experiments were performed with biologically independent samples (Fan et al., 2006). These three microarray datasets were generated with the use of the GPL570 Affymetrix GeneChip Human Genome U133 Plus 2.0 Array. The comparison data of each dataset were uploaded as the Supplementary Tables 1–3.

### Data Processing of Differentially Expressed Genes

The up-regulated genes when ER is activated by estrogen and the down-regulated genes when ER is silenced may be genes positively regulated by ER. In contrast, the down-regulated genes when ER is activated by estrogen and the up-regulated genes when ER is silenced may be genes that are negatively regulated by ER. Three gene expression datasets (GSE11324, GSE27473, and GSE5840) containing paired gene expression data were used for the comparative analyses. DEGs in the three datasets were identified with the GEO2R online tool (Davis and Meltzer, 2007) using a adjust *P* < 0.05. Values for log_2_FC > 0.5 in GSE11324 and GSE5840 (comparison data for wild-type MCF7 cells) and log_2_FC < −0.5 in GSE27473 were considered to indicate genes positively regulated by ER, whereas log_2_FC < −0.5 in GSE11324 and GSE5840 (comparison data for wild-type MCF7 cells) and log_2_FC > 0.5 in GSE27473 were considered to indicate genes negatively regulated by ER. Next, the raw data were plotted with the Venn diagram software^1^, and the common DEGs among the three datasets above were obtained.

### GO and Pathway Enrichment Analyses

Gene ontology analysis is commonly used to define genes and their expression products to identify unique biological properties of high-throughput transcriptome or genome data (Ashburner et al., 2000). Kyoto Encyclopedia of Gene and Genome (KEGG) is a collection of databases dealing with genomes, diseases, biological pathways, drugs, and chemical materials (Du et al., 2014). The Database for Annotation, Visualization, and Integrated Discovery (DAVID^2^) is an online bioinformatics tool for functional annotation of large numbers of genes or proteins (Huang da et al., 2009). We used DAVID to perform GO and KEGG analyses of the list of DEGs we had identified, in terms of molecular function (MF), cellular component (CC), and biological process (BP); the terms with a *P* < 0.05 would be taken into consideration.

### PPI Network and Modular Analyses

We used the online tool, STRING (Search Tool for the Retrieval of Interacting Genes^3^) (Szklarczyk et al., 2015), to extract PPI information from the list of identified DEGs. Cytoscape (Shannon et al., 2003) was used to visualize these DEGs with STRING, and the PPI network was analyzed with the MCODE (Molecular Complex Detection) plug-in to identify the significant gene clusters (degree cutoff = 2, maximum depth = 100, k-core = 2, and node score cutoff = 0.2). MCODE is a graph theoretic clustering algorithm that detects densely connected regions in PPI networks that may represent molecular complexes, and larger, more dense complexes have higher MCODE scores (Bader and Hogue, 2003).

### Relapse-Free Survival Analyses

We used the Kaplan–Meier plotter online tool^4^ to explore the RFS of patients with ER-positive/HER2-negative breast cancer who had undergone endocrine therapies only. It was a specific group of patients who were not interfered with by other therapeutic strategies, and the recurrence of breast cancer was more likely due to endocrine therapy resistance. The Kaplan–Meier plotter for breast cancer is commonly used to assess the effect of genes on survival; the background database was established using gene expression data and survival information of breast cancer patients obtained from the GEO database (Gyorffy et al., 2010). The median of each gene expression probe as a cutoff value splitting the patient into high- and low-expression groups, and when a logrank *P* < 0.05 was considered to have the RFS difference. In this way, the candidate genes related to endocrine therapy resistance can be screened.

### Expression Patterns Analyses of the RFS-Related Genes

We compared the gene expression data of the RFS-related genes in GSE5840 dataset, which contains the gene expression information of wild-type, tamoxifen-resistant, and fulvestrant-resistant MCF7 cells treated with estrogen. Differences among groups were analyzed by the Student’s *t*-test, one-way analysis of variance and least significant difference, and Student–Newman–Keuls *post hoc* test. *P* < 0.05 was considered to be statistically significant.

### Workflow

**FIGURE 1:**
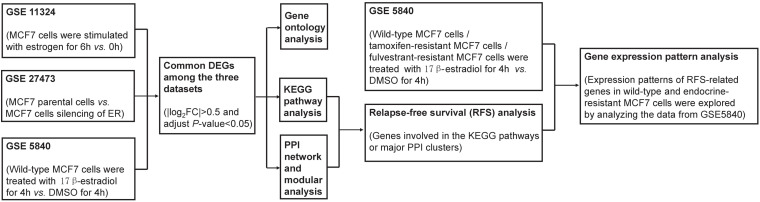
The diagram of the workflow and datasets.

The diagram of the workflow and datasets is shown in Figure 1.

## Results

### Identification of Genes Regulated by ER in MCF7 Cells

**FIGURE 2:**
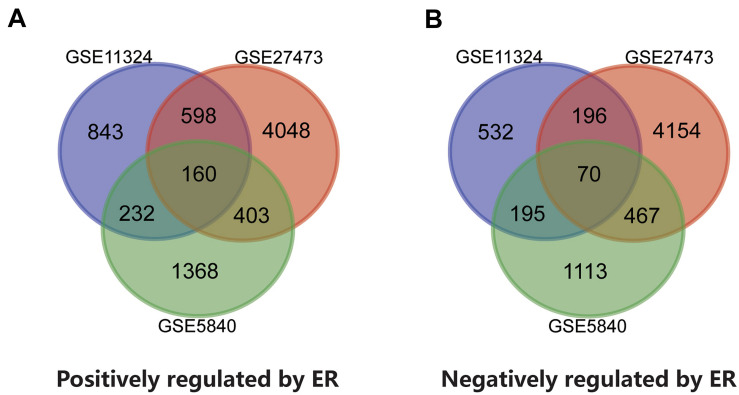
A total of 230 common differentially expressed genes (DEGs) were identified among the three datasets (GSE11324, GSE27473, and GSE5840) using Venn diagrams. **(A)** There were 160 genes positively regulated by ER. **(B)** There were 70 genes negatively regulated by ER. Different colors represent different datasets.

Three datasets containing paired gene expression data, namely, GSE11324, GSE27473, and GSE5840, were analyzed in the present study. The up-regulated genes when ER is activated by estrogen and the down-regulated genes when ER is silenced may be genes positively regulated by ER. In contrast, the down-regulated genes when ER is activated by estrogen and the up-regulated genes when ER is silenced may be genes that are negatively regulated by ER. Using the GEO2R online tool, we identified 1,833, 5,209, and 2,163 genes meeting the standard to be positively regulated by ER in GSE11324, GSE27473, and GSE5840, respectively. Meanwhile, there were 993, 4,887, and 1,845 genes to be negatively regulated by ER in GSE11324, GSE27473, and GSE5840, respectively. Venn diagrams were used to obtain the common DEGs among the three datasets. There were 230 common DEGs associated with ER activation or silence, comprising 160 genes positively regulated by ER and 70 genes negatively regulated by ER (Table 1 and Figure 2).

**TABLE 1:** List of the 230 common DEGs in response to ER activation or silence identified from three datasets.

**ER regulating genes**	**Genes name**
Positively regulated by ER	*PNPT1*, *MREG*, *MAX*, *MED13L*, *CAMTA1*, *THADA*, *DDX18*, *CENPU*, *MAK16*, *TPBG*, *SLC9A3R1*, *FKBP4*, *LONRF2*, *KRT15*, *MAD2L1*, *IFITM10*, *SYTL5*, *JAK2*, *C6orf141*, *ELF1*, *MAST4*, *CA12*, *ZC3H14*, *ADCY1*, *SLC25A24*, *SLC22A5*, *BRI3BP*, *LOC441155///ZC3H11A*, *NKAIN1*, *SIAH2*, *CALM3///CALM2///CALM1*, *POLR1B*, *CHML*, *TMPRSS3*, *ZBTB21*, *RPRD1A*, *EIF3J*, *THBS1*, *CALCR*, *SLC26A2*, *PGR*, *TMEM64*, *ZDHHC21*, *ZMYM2*, *PIK3R1*, *PTRH2*, *SKIL*, *PLA2G12A*, *RHOBTB1*, *GEMIN5*, *AFF3*, *MYB*, *MPPED2*, *NUFIP2*, *RPP40*, *DGKH*, *KRT13*, *DCLRE1B*, *PUS1*, *RET*, *WWC1*, *SMG1P5///BOLA2///SMG1P2*, *KIF21A*, *NOP16*, *LRPPRC*, *IL17RB*, *EGR3*, *SLC7A2*, *NIFK*, *THRIL///BRI3BP*, *USP31*, *SNRPA1*, *DSCAM*, *AKAP1*, *EIF5*, *NBPF4*, *TBC1D30*, *HSPA9*, *GTPBP4*, *SLC1A1*, *PUS7*, *LOC101928589///TMEM164*, *CXCL12*, *PPP4R3A*, *MLLT10*, *BAZ1A*, *PPAT*, *C1orf226*, *LOC101060386///BOLA2///SMG1*, *PTP4A1*, *SNX24*, *STC2*, *SGO2*, *ADCY9*, *MDM4*, *LOC101060275///NPIPA5///NPIPB5///NPIPB11///LOC613037///NPIPB4///NPIPB3*, *TTC3P1///TTC3*, *CAND1*, *CHRNA5*, *MTRR*, *MSMB*, *ZNF75A*, *ABHD2*, *PKIB*, *AMMECR1*, *GABPB1-AS1*, *BARD1*, *CNOT7*, *MIR4657///PURB*, *TPD52L1*, *SLC19A1*, *GNA13*, *GLCCI1*, *ZFX*, *NUP35*, *HDDC2*, *DMXL2*, *PIP4K2A*, *ELOVL2*, *MED13*, *TRNT1*, *GSPT1*, *DGKE*, *AGPAT5*, *MRPS25*, *C5orf22*, *RSL1D1*, *SLC16A1*, *SMIM13*, *CYP1B1*, *SLC19A2*, *AGR3*, *METTL8*, *RFC3*, *GREB1*, *MBOAT1*, *NRIP1*, *RAPGEFL1*, *FAM208B*, *ANAPC7*, *NBPF1*, *SOX3*, *TPM1*, *TFRC*, *KPNA1*, *RLN2*, *RARA*, *RASGRP1*, *MGA*, *TET2*, *SRSF1*, *PCDH10*, *PA2G4*, *RAB30*, *DEPTOR*, *PDZK1*, *IGFBP4*, *CHAC2*, *ONECUT2*, *SCAF11*
Negatively regulated by ER	*HIC2*, *TMEM42*, *SPTBN1*, *CYP1A1*, *AHNAK*, *SOX9*, *PLEKHM3*, *JRK*, *TPGS1*, *SOCS3, LOC100288911*, *LOC100996760*, *PBXIP1*, *AJUBA*, *SNORA11E///SNORA11D///MAGED4///MAGED4B*, *MIR4746///UBXN6*, *KLHL5*, *IL1R1*, *ABTB1*, *CTTN*, *RGS3*, *CHEK1*, *FZD2*, *PARVA*, *TP53INP2*, *KIAA1217*, *CAPG*, *PCGF2*, *C10orf10*, *CTGF*, *GLMP*, *DDIT4*, *LYN*, *BRI3*, *SERPINB1*, *ING4*, *PANX2*, *CDKN1C*, *HEMK1*, *TBC1D2*, *SHISA2*, *ENC1*, *LMNA*, *FBXO32*, *SH3BP4*, *HS3ST1*, *GATA6*, *ORAI3*, *APOBEC3B*, *PXN*, *NEDD4L*, *BCAR3*, *LOC102725292///DTX2*, *EXT1*, *NGEF*, *KRT7, ALDH1A3*, *UBALD2*, *C11orf96*, *SLC17A5*, *INO80C*, *TLDC1*, *TAGLN*, *IFIT1*, *PLEKHM1*, *PARP10*, *BCL2L1*, *MIR4800///MXD4*, *NUMA1*, *GPR39*

### GO and KEGG Pathway Analyses for ER Regulating Genes

**FIGURE 3:**
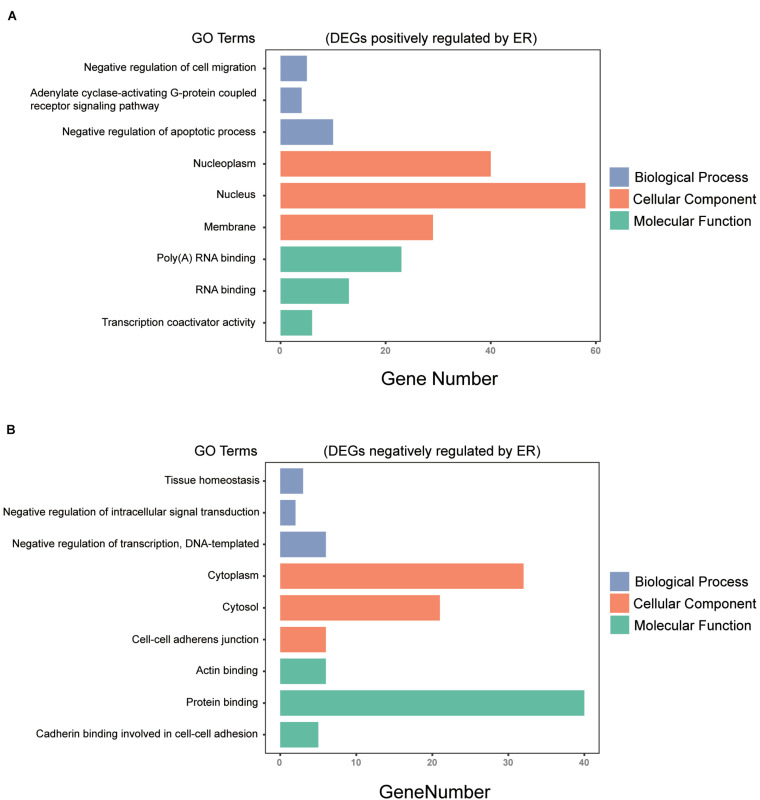
Gene ontology (GO) analysis of the common DEGs among the three breast cancer datasets. **(A)** DEGs positively regulated by ER were analyzed by GO enrichment, the top 3 in *P*-value ranking (sorting from small to large) in biological process (BP), cellular component (CC), and molecular function (MF). **(B)** DEGs negatively regulated by ER were analyzed by GO enrichment, the top 3 in *P*-value ranking (sorting from small to large) in BP, CC, and MF (*P* < 0.05).

All 230 common DEGs were analyzed with the DAVID functional annotation tool, which included the functional categories of BP, CC, MF, and KEGG pathways. For BP, the genes positively regulated by ER mainly showed enrichment for the negative regulation of apoptotic process, negative regulation of cell migration, and adenylate cyclase-activating G-protein-coupled receptor signaling pathway (*P* < 0.05). The genes negatively regulated by ER mainly showed enrichment for the negative regulation of transcription, tissue homeostasis, and negative regulation of intracellular signal transduction (*P* < 0.05). For CC, the ER positively regulating genes were mainly enriched for the nucleoplasm, nucleolus, and membrane (*P* < 0.05). Moreover, the ER negatively regulating genes were mainly enriched for the cytoplasm, cytosol, and cell–cell adherens junction (*P* < 0.05). For MF, the ER positively regulating genes were mainly enriched for RNA binding and transcription coactivator activity (*P* < 0.05). The ER negatively regulating genes were mainly enriched for protein binding, actin binding, and cadherin binding involved in cell–cell adhesion (*P* < 0.05) (Figure 3).

Kyoto Encyclopedia of Gene and Genome pathway enrichment analysis demonstrated that ER might regulate genes along pathways associated with cancer, progesterone-mediated oocyte maturation, RNA transport, glycerophospholipid and glycerolipid metabolism, oocyte meiosis, platelet activation, phosphatidylinositol signaling system, estrogen signaling pathway, and nuclear factor κB (NF-κB) signaling pathway (*P* < 0.05). The genes involved in each pathway are listed in Table 2.

**TABLE 2:** KEGG pathway analysis of the common DEGs among the three datasets.

**Term**	**Count**	***P*-value**	**Genes**
hsa04914: progesterone-mediated oocyte maturation	6	7.80E-04	*PGR*, *ADCY1*, *MAD2L1*, *ADCY9*, *ANAPC7*, *PIK3R1*
hsa00564: glycerophospholipid metabolism	5	0.008	*AGPAT5*, *DGKE*, *PLA2G12A*, *MBOAT1*, *DGKH*
hsa00561: glycerolipid metabolism	4	0.013	*AGPAT5*, *DGKE*, *MBOAT1*, *DGKH*
hsa04114: oocyte meiosis	5	0.014	*PGR*, *ADCY1*, *MAD2L1*, *ADCY9*, *ANAPC7*
hsa03013: RNA transport	6	0.015	*TRNT1*, *EIF5*, *EIF3J*, *NUP35*, *RPP40*, *GEMIN5*
hsa05200: pathways in cancer	9	0.016	*GNA13*, *MAX*, *ADCY1*, *RET*, *ADCY9*, *RASGRP1*, *RARA*, *CXCL12*, *PIK3R1*
hsa04611: platelet activation	5	0.024	*GNA13*, *ADCY1*, *ADCY9*, *RASGRP1*, *PIK3R1*
hsa04064: NF-κB signaling pathway	3	0.045	*IL1R1*, *LYN*, *BCL2L1*
hsa04070: phosphatidylinositol signaling system	4	0.049	*DGKE*, *DGKH*, *PIP4K2A*, *PIK3R1*
hsa04915: estrogen signaling pathway	4	0.049	*ADCY1*, *ADCY9*, *FKBP4*, *PIK3R1*

### PPI Network and Modular Analyses

**FIGURE 4:**
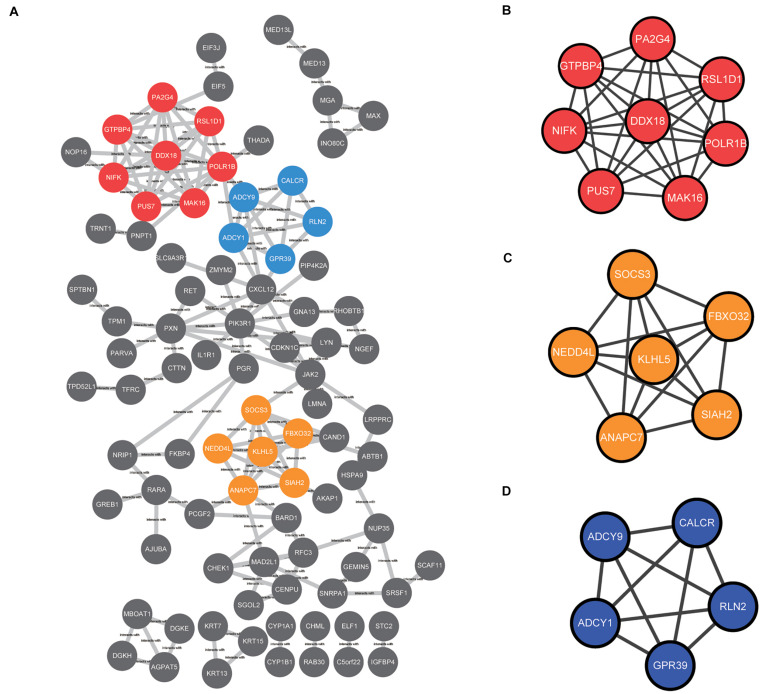
The protein–protein interaction (PPI) network among the common DEGs was constructed and then subjected to modular analysis with Cytoscape software and the MCODE plug-in (degree cutoff = 2, node score cutoff = 0.2, k-core = 2, and maximum depth = 100). **(A)** The PPI network complex, including 89 nodes and 149 edges. The nodes meant proteins; the edges meant the interaction of proteins; three major clusters were identified by MCODE plug-in and labeled by red, orange, and blue colors. **(B)** The cluster with the top MCODE score (score = 8.0) contained 8 nodes and 28 edges labeled red. **(C)** The cluster with the second-highest MCODE score (score = 6.0) contained 6 nodes and 15 edges labeled orange. **(D)** The cluster with the third-highest MCODE score (score = 5.0) contained 5 nodes and 10 edges labeled blue.

The common DEGs identified among the three datasets were imported into STRING online tool to analyze the PPI network. A total of 89 of the 230 DEGs were contained into the PPI network complex, including 89 nodes and 149 edges (Figure 4A). Moreover, we applied MCODE, a plug-in of the Cytoscape software for further analysis. Three major clusters were identified according to the MCODE score. The cluster with the top MCODE score (score = 8.0) contained 8 nodes and 28 edges: *PUS7*, *POLR1B*, *MAK16*, *RSL1D1*, *NIFK*, *PA2G4*, *GTPBP4*, *DDX18* (Figure 4B). Followed by the cluster (MCODE score = 6.0), which contained 6 nodes and 15 edges: *NEDD4L*, *SOCS3*, *SIAH2*, *KLHL5*, *ANAPC7*, and,*FBXO32* (Figure 4C). The cluster with the third-highest MCODE score (score = 5.0) included 5 nodes and 10 edges: *ADCY9*, *ADCY1*, *GPR39*, *CALCR*, and,*RLN2* (Figure 4D).

### Relapse-Free Survival Analyses

**FIGURE 5:**
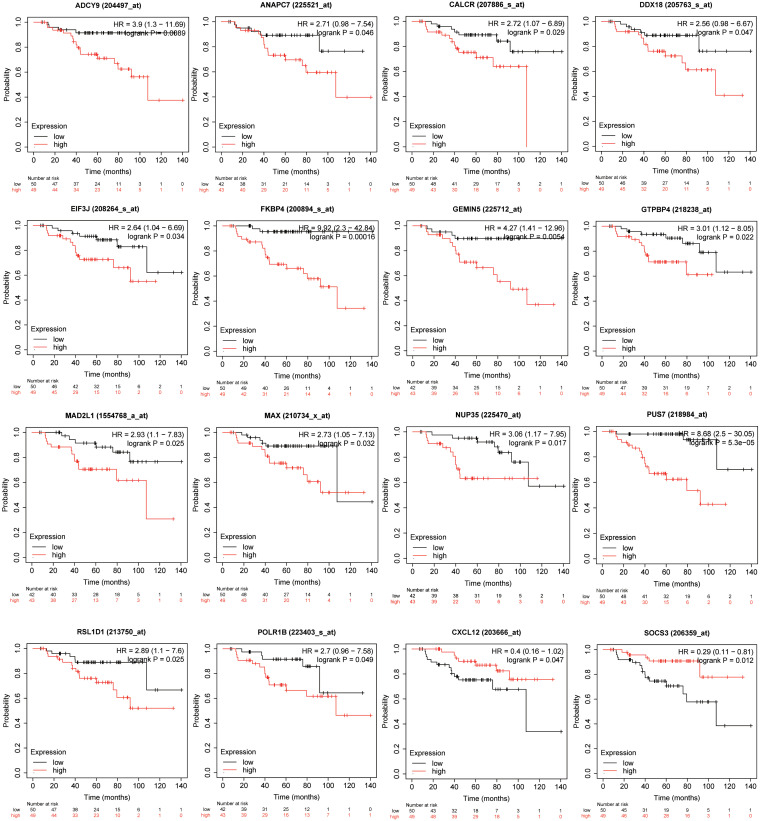
Kaplan–Meier plotter was used to analyze associations between the expressions of the genes involved in the KEGG pathways or major PPI clusters and relapse-free survival (RFS) in patients with ER-positive/HER2-negative breast cancer who had undergone endocrine therapies only. This analysis identified 16 of 44 genes as being associated with a significantly different RFS (*P* < 0.05).

Genes involved in the KEGG pathways or major PPI clusters were imported into the Kaplan–Meier plotter online tool to analyze associations between the genes’ expression and RFS from patients with ER-positive/HER2-negative breast cancer who had undergone endocrine therapies only. It was a specific group of patients who were not interfered with by other therapeutic strategies, and the recurrence of breast cancer was more likely due to endocrine therapy resistance. Among these, the expression of 16 genes was found to be significantly associated with RFS (*P* < 0.05), whereas the other 28 genes had no significant associations (Table 3). The 16 genes were *ADCY9*, *ANAPC7*, *CALCR*, *CXCL12*, *DDX18*, *EIF3J*, *FKBP4*, *GEMIN5*, *GTPBP4*, *MAD2L1*, *MAX*, *NUP35*, *POLR1B*, *PUS7*, *RSL1D1*, and *SOCS3* (Figure 5). In this way, the candidate genes related to endocrine therapy resistance could be screened.

**TABLE 3:** The prognostic value (RFS) of the selected genes in patients with ER-positive/HER2-negative breast cancer who had undergone endocrine therapies only.

**Category**	**Genes**
Genes significantly associated with RFS (*P* < 0.05)	*ADCY9*, *ANAPC7*, *CALCR*, *CXCL12*, *DDX18*, *EIF3J*, *FKBP4*, *GEMIN5*, *GTPBP4*, *MAD2L1*, *MAX*, *NUP35*, *POLR1B*, *PUS7*, *RSL1D1*, *SOCS3*
No significant associations with RFS (*P* > 0.05)	*ADCY1*, *AGPAT5*, *BCL2L1*, *DGKE*, *DGKH*, *EIF5*, *FBXO32*, *GNA13*, *GPR39*, *IL1R1*, *KLHL5*, *LYN*, *MAK16*, *MBOAT1*, *NEDD4L*, *NIFK*, *PA2G4*, *PGR*, *PIK3R1*, *PIP4K2A*, *PLA2G12A*, *RARA*, *RASGRP1*, *RET*, *RLN2*, *RPP40*, *SIAH2*, *TRNT1*

*These genes were involved in the KEGG pathways or major PPI clusters. RFS, relapse-free survival.*

### Expression Patterns Analyses of the Screened Genes Among Wild-Type, Tamoxifen-Resistant, and Fulvestrant-Resistant MCF7 Cells

**FIGURE 6:**
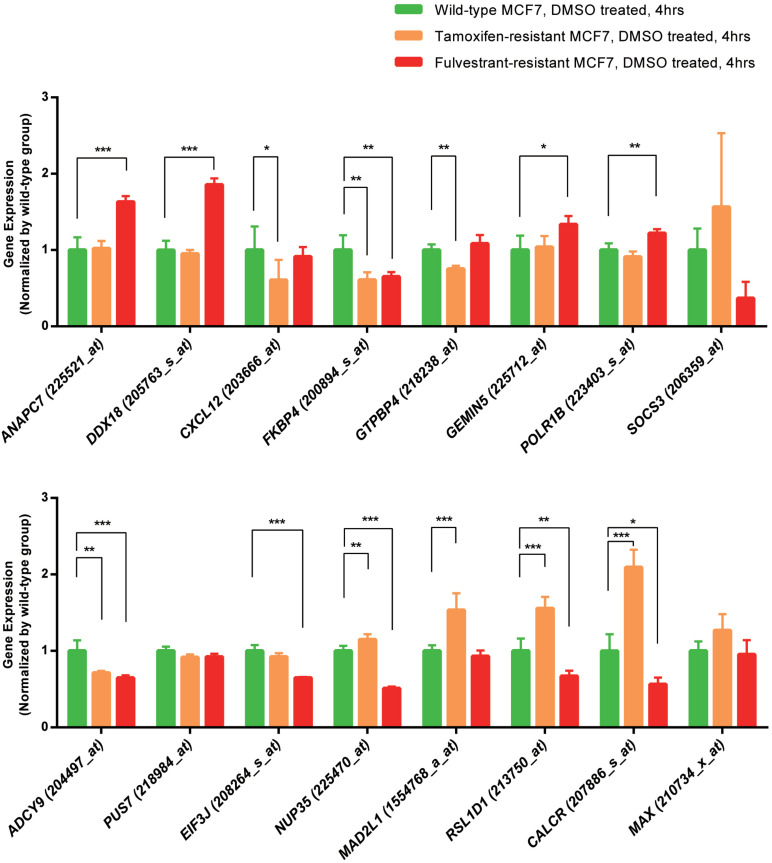
The comparisons of the screened RFS-related genes’ expression among the wild-type, tamoxifen-resistant, and fulvestrant-resistant MCF7 cells (treated cells with DMSO) in GSE5840 dataset (*P < 0.05, **P < 0.01, ***P < 0.001).

**FIGURE 7:**
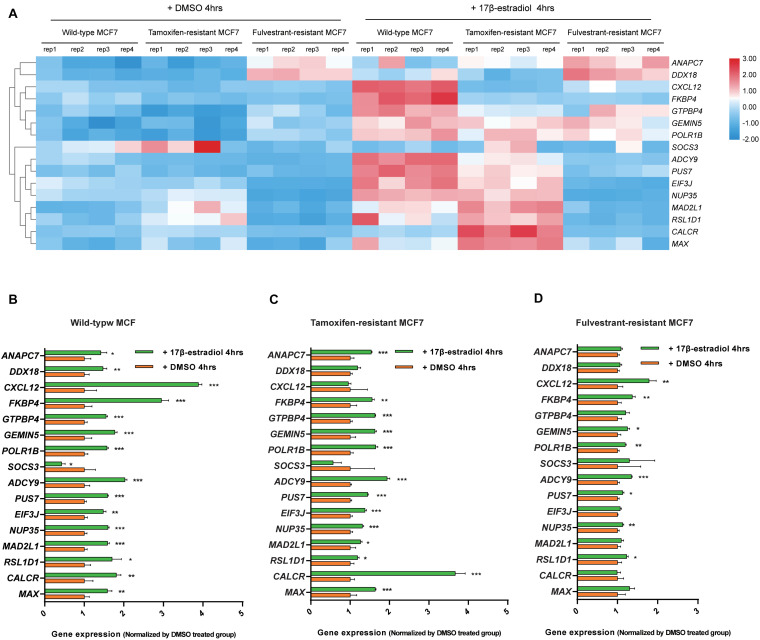
Analyses of the RFS-related genes’ expression data among the wild-type, tamoxifen-resistant, and fulvestrant-resistant MCF7 cells under the treatment of estrogen or DMSO in GSE5840 dataset. **(A)** Heat map of the gene expression data in six groups (wild-type MCF7 cells treated with 17β-estradiol or DMSO, tamoxifen-resistant MCF7 cells treated with 17β-estradiol or DMSO, fulvestrant-resistant MCF7 cells treated with 17β-estradiol or DMSO). **(B)** The comparisons of gene expression between the 17β-estradiol treatment group and negative control (treated with DMSO) for wild-type MCF7 cells. **(C)** The comparisons of gene expression between the 17β-estradiol treatment group and negative control for tamoxifen-resistant MCF7 cells. **(D)** The comparisons of gene expression between the 17β-estradiol treatment group and negative control for fulvestrant-resistant MCF7 cells (*P < 0.05, **P < 0.01, ***P < 0.001).

We analyzed the expression data of the 16 RFS-related genes in GSE5840 dataset, which contains comparison data, including wild-type MCF7 cells treated with 17β-estradiol versus negative control [dimethyl sulfoxide (DMSO)], tamoxifen-resistant MCF7 cells treated with 17β-estradiol versus negative control (DMSO), and fulvestrant-resistant MCF7 cells treated with 17β-estradiol versus negative control (DMSO). First, we compared the gene expression data among wild-type, tamoxifen-resistant, and fulvestrant-resistant MCF7 cells in the negative control groups. There were different gene expression patterns among the three cell lines. For instance, the genes such as *ANAPC7* and *DDX18* were significantly up-regulated (log_2_FC > 0.5 and *P* < 0.05) in the fulvestrant-resistant cells, and the *MAD2L1*, *RSL1D1*, and *CALCR* were significantly up-regulated (log_2_FC > 0.5 and *P* < 0.05) in the tamoxifen-resistant cells**;** all the comparison results are shown in Figure 6. Then, we analyzed the gene expression data among the three kinds of cell lines treated with estrogen or DMSO (negative control) (Figure 7). Comparing with wild-type cells, nearly all of the genes’ expression levels changed less in fulvestrant-resistant cells when treated with estrogen. However, in tamoxifen-resistant cells, the change patterns of gene expression under the estrogen treatment were similar to that of wild-type cells except for *DDX18*, *CXCL12*, *FKBP4*, *NUP35*, *MAD2L1*, *RSL1D1* (changed less), and *CALCR* (changed more).

## Discussion

Estrogen and ER play essential roles in the development and progression of ER-positive breast cancer. At the same time, the phenomenon of endocrine therapy resistance occurs frequently and complicates patient management (Rani et al., 2019). However, the regulatory network of the ER has not yet been fully elucidated (Bhuva et al., 2019). The present study used bioinformatics methods to interrogate three gene expression datasets from MCF7 cells treated with estrogen, MCF7 cells subjected to the silencing of the ER, and tamoxifen- and fulvestrant-resistant MCF7 cells treated with 17β-estradiol, respectively. By using these datasets, the function of the ER could be explored from different perspectives. Not only did we perform GO and KEGG pathway analyses, but also screened RFS-related genes and explored their expression pattern changes in tamoxifen- and fulvestrant-resistant cells.

There are two classes of ER, including nuclear ER (intracellular receptor) and membrane ER (mostly G-protein-coupled receptor) (Razandi et al., 1999). For nuclear ER (ERα and ERβ), once activated by estrogen, the ER can translocate into the nucleus and regulate the activity of different genes (Yasar et al., 2017). In the present study, GO and KEGG analyses showed that ER positively regulating genes were mainly localized in the nucleoplasm, nucleolus, and membrane, involving biological processes such as negative regulation of apoptotic process and adenylate cyclase-activating G-protein-coupled receptors signaling pathway, which were corresponding to the two forms of ER as the nuclear receptor and the membrane receptor. The molecular functions such as RNA binding and transcription coactivator activity could create conditions for genetic transcription and cell proliferation. Meanwhile, the biological processes for ER negatively regulating genes were negative regulation of transcription and negative regulation of intracellular signal transduction, further reflecting the main functions of the two forms of ER.

DEGs involved in the KEGG pathways or major PPI clusters were analyzed with Kaplan–Meier plotter to determine whether there were any associations between these genes and the RFS of patients with ER-positive/HER2-negative breast cancer who had undergone endocrine therapies only. It is a specific group of patients who were not interfered with by other therapeutic strategies; such analysis may best reflect the interplay between ER-regulated genes and the development of endocrine resistance. *ADCY9*, *ANAPC7*, *CALCR*, *CXCL12*, *DDX18*, *EIF3J*, *FKBP4*, *GEMIN5*, *GTPBP4*, *MAD2L1*, *MAX*, *NUP35*, *POLR1B*, *PUS7*, *RSL1D1*, and *SOCS3* were found to be significantly associated with RFS. We subsequently analyzed the expression patterns of these genes among wild-type, tamoxifen-resistant, and fulvestrant-resistant MCF7 cells. Interestingly enough, nearly all of the genes’ expression level changed less when treated with estrogen in fulvestrant-resistant cells compared with the wild type, indicating that the function of ER is weakened or disappeared in fulvestrant-resistant MCF7 cells. Fulvestrant is a selective ER degrader; it works by binding to and destabilizing the ERs (Lai and Crews, 2017). If ERs ceased to function in breast cancer cells, fulvestrant resistance would be occurring. *ANAPC7* and *DDX18* were ER positively regulating genes in wild-type MCF7 cells. However, they were significantly up-regulated in the fulvestrant-resistant cells without estrogen treatment, speculating that these genes may play essential roles in promoting the survival of fulvestrant-resistant breast cancer cells.

When tamoxifen-resistant cells were treated with estrogen, the expression change patterns of many genes were similar to that of wild-type cells, suggesting that the function of ER exists in tamoxifen-resistant MCF7 cells. As the selective ER modulator, tamoxifen blocks the effects of estrogen by attaching to the ERs in breast cells (Goodsell, 2002). The existence of functional ERs explains why fulvestrant treatment may still be effective when tamoxifen resistance occurs. Meanwhile, in the negative control groups, *MAD2L1*, *RSL1D1*, and *CALCR* were found to be significantly up-regulated in the tamoxifen-resistant cells compared with wild-type cells. It was speculated that these genes might play crucial roles in tamoxifen-resistant breast cancer cells.

Dead-box RNA helicase 18 (DDX18) is an essential factor in cell cycle progression in zebrafish hematopoietic cells and is mutated in some patients with acute myeloid leukemia (Payne et al., 2011). Redmond et al. (2015) identified DDX18 as a novel driver of endocrine resistance in breast cancer. They found a significant inhibition of the proliferation of endocrine-resistant cell lines based on an increased G1 phase cell population when *DDX18* was silenced. By analyzing clinical samples, they discovered that the mRNA levels of *DDX18* were significantly correlated with poor prognosis in breast cancer patients (Redmond et al., 2015). It is consistent with our results. High expression of *DDX18* was correlated with significantly worse RFS in ER-positive/HER2-negative breast cancer patients who had undergone endocrine therapies only, and the expression level of *DDX18* was continuously high in the fulvestrant-resistant breast cancer cells. Naorem et al. (2019) investigated six microarray datasets from the Gene Expression Omnibus consisting of 405 triple-negative breast cancer (ER/PR/HER2-negative, TNBC) and 463 non-TNBC samples and identified 1,075 DEGs; *DDX18* was 1 of 12 up-regulated genes identified as essential by a machine learning-based feature selection method (Naorem et al., 2019). In the present study, we found that the function of the ER is weakened or disappeared in fulvestrant-resistant MCF7 cells, suggesting that these cells might possess the characteristics of TNBC. With the *DDX18* exhibits significantly higher expression in TNBC and is no longer regulated by ER in fulvestrant-resistant MCF7 cells. These phenomena suggest that *DDX18* might play a vital role in endocrine therapy resistance and that its activity might confer characteristics of TNBC onto ER-positive breast cancer.

*ANAPC7* gene encodes a tetratricopeptide repeat-containing component of the anaphase-promoting complex/cyclosome (APC/C), a multisubunit ubiquitin ligase that is essential for mitosis by targeting a number of cell cycle regulators such as cyclin B1 and promoting timely degradation (Wild et al., 2018). Reducing the activity of APC/C delays mitotic progression, whereas APC/C activity loss is lethal (Magnuson and Epstein, 1984; Wirth et al., 2004). In our study, high expression of *ANAPC7* was correlated with significantly worse RFS in the specific cohort of breast cancer patients, and the expression level of *ANAPC7* was continuously high in the fulvestrant-resistant breast cancer cells. Considering the vital function of APC/C in eukaryotic cells mitosis (Pines, 2011), *ANAPC7* might play an essential role in fulvestrant resistance, and ANAPC7 (anaphase-promoting complex subunit 7) was expected to be a novel biomarker or therapeutic target for fulvestrant-resistant breast cancer.

Other up-regulated genes such as *GEMIN5* and *POLR1B* (values of log_2_FC were between 0 and 0.5, and *P* < 0.05) might also be associated with fulvestrant resistance. Gem nuclear organelle associated protein 5 (Gemin5) is an RNA-binding protein that was identified as a component of the survival of motor neurons (SMN) complex (Pineiro et al., 2015). The SMN complex plays a critical role in mRNA splicing and may mediate the assembly and transport of other classes of ribonucleoproteins (Massenet et al., 2002). *GEMIN5* codes Gemin5*;* alteration of *GEMIN5* expression may play a role in alternative mRNA splicing and tumor cell motility (Lee et al., 2008). Recently, a study reported that *POLR1B* is up-regulated in non-small cell lung cancer and may serve an important modulator of lung cancer cell proliferation (Yang et al., 2020). Our research added to the understanding of *GEMIN5* and *POLR1B* genes, and they might serve as biomarkers for fulvestrant resistance in ER-positive breast cancer.

In tamoxifen-resistant cells, *MAD2L1*, *RSL1D1*, and *CALCR* were found to be significantly up-regulated compared with the wild-type cells. MAD2L1 (mitotic arrest deficient 2 like 1) plays an essential role in supervising chromosome segregation as the component of spindle checkpoint during mitosis (Guo et al., 2010). Studies showed that *MAD2L1* presented overexpression in breast cancer and was significantly associated with higher clinical stage, higher histological grade, aggressive tumors, and worse disease-free survival (Wang et al., 2015; Zhu et al., 2017). RSL1D1 (ribosomal L1 domain containing 1) is a nucleolar protein that has been demonstrated to delay cellular senescence and serve as an independent prognostic factor in prostate cancer (Li et al., 2016). *CALCR* (coding calcitonin receptor) has similar gene expression patterns to *MAD2L1* and *RSL1D1* in tamoxifen-resistant MCF7 cells; the expression of these genes was all correlated with worse RFS in ER-positive/HER2-negative breast cancer patients who had undergone endocrine therapies only. In addition, compared with the wild-type MCF7 cells, the expression level of *MAD2L1*, *RSL1D1*, and *CALCR* was significantly up-regulated in tamoxifen-resistant cells even without estrogen treatment. However, there has not been sufficient research on the relationship between the proteins coded by these genes and endocrine therapy resistance in breast cancer. Therefore, MAD2L1, RSL1D1, and CALCR might be promising candidates for further research in endocrine therapy-resistant breast cancer as potential therapeutic targets or prognostic markers.

By identifying the significant genes interacting with the ER and their expression pattern changing when tamoxifen or fulvestrant resistance occurs, we not only can obtain a better understanding of this essential receptor’s regulatory network but also can speculate some possible mechanisms of endocrine resistance. The main assumptions are as follows: (1) in fulvestrant-resistant breast cancer cells, the expression change of most estrogen-regulated genes was not evident under estrogen treatment, indicating that the function of ER is weakened or disappeared in fulvestrant-resistant cells. Fulvestrant works by destabilizing the ERs, and if ERs ceased to function in breast cancer cells, fulvestrant resistance would occur. (2) *DDX18* and *ANAPC7* might play a vital role in fulvestrant-resistant breast cancer cells because of their crucial functions in cell cycle progression and eukaryotic cells mitosis (Pines, 2011; Redmond et al., 2015) and continuous overexpression in the fulvestrant-resistant breast cancer cells. So they were expected to be novel biomarkers or therapeutic targets for further study. (3) In tamoxifen-resistant breast cancer cells, the expression changes of many genes were similar to wild-type cells under estrogen treatment, suggesting that the ER’s function exists in tamoxifen-resistant cells, and explaining that fulvestrant treatment might still be valid when tamoxifen resistance occurs. (4) Some genes such as *MAD2L1*, *RSL1D1*, and *CALCR* were found to be significantly up-regulated in the tamoxifen-resistant cells, indicating that tamoxifen might not completely block the estrogen signaling in tamoxifen-resistant breast cancer cells. These highly expressed genes would be potential biomarkers for tamoxifen resistance, and suppression of them might be an important direction to overcome tamoxifen resistance in the future.

In summary, this bioinformatics analysis study identified the significant genes regulated by ER in ER-positive breast cancer cells. As endocrine resistance in ER-positive breast cancer is likely to be attributable to the abnormal regulation of ER network, the expression pattern changes of these genes were explored in tamoxifen- and fulvestrant-resistant breast cancer cells. Although these predictions need to be further validated, the present study provided useful insights regarding potential biomarkers and the pathomechanisms of ER-positive breast cancer resistant to endocrine therapy.

## Data Availability Statement

The datasets generated for this study can be found in the GEO database, GSE11324, GSE27473, GSE5840.

## Author Contributions

JW, YF, and ZW conceived the idea and revised the final manuscript. RC and LQ designed the work, analyzed the data, and drafted the manuscript. XK interpreted the data and revised the manuscript. All authors read and approved the final manuscript. They agreed to be accountable for all aspects of the work in ensuring that questions related to the accuracy or integrity of any part of the work were appropriately investigated and resolved.

## Conflict of Interest

The authors declare that the research was conducted in the absence of any commercial or financial relationships that could be construed as a potential conflict of interest.

## Footnotes

1 http://bioinformatics.psb.ugent.be/webtools/Venn/

2 https://david.ncifcrf.gov/

3 https://string-db.org/

4 https://kmplot.com/analysis/

## Supplementary Material

SUPPLEMENTARY TABLE 1 The comparison results from GSE11324 dataset (MCF7 cells were stimulated with estrogen for 6 vs. 0 h).

SUPPLEMENTARY TABLE 2 The comparison results from GSE27473 dataset (MCF7 parental cells vs. MCF7 cells silencing of ER).

SUPPLEMENTARY TABLE 3 The comparison results from GSE5840 dataset (wild-type MCF7 cells were treated with 17β-estradiol for 4 h vs. DMSO for 4 h).

## References

[B1] Al Saleh S. Al Mulla F. Luqmani Y. A. (2011). Estrogen receptor silencing induces epithelial to mesenchymal transition in human breast cancer cells. *PLoS One* 6:e20610. 10.1371/journal.pone.0020610 21713035 PMC3119661

[B2] Ashburner M. Ball C. A. Blake J. A. Botstein D. Butler H. Cherry J. M. (2000). Gene ontology: tool for the unification of biology. The gene ontology consortium. *Nat. Genet.* 25 25–29. 10802651 10.1038/75556 PMC3037419

[B3] Bader G. D. Hogue C. W. (2003). An automated method for finding molecular complexes in large protein interaction networks. *BMC Bioinform.* 4:2. 10.1186/1471-2105-4-2 12525261 PMC149346

[B4] Bhuva D. D. Cursons J. Smyth G. K. Davis M. J. (2019). Differential co-expression-based detection of conditional relationships in transcriptional data: comparative analysis and application to breast cancer. *Genome Biol.* 20:236. 10.1186/s13059-019-1851-8 PMC6857226 31727119

[B5] Carroll J. S. Meyer C. A. Song J. Li W. Geistlinger T. R. Eeckhoute J. (2006). Genome-wide analysis of estrogen receptor binding sites. *Nat. Genet.* 38 1289–1297. 17013392 10.1038/ng1901

[B6] Clarke R. Tyson J. J. Dixon J. M. (2015). Endocrine resistance in breast cancer–an overview and update. *Mol. Cell. Endocrinol.* 418(Pt 3), 220–234. 10.1016/j.mce.2015.09.035 26455641 PMC4684757

[B7] Davis S. Meltzer P. S. (2007). GEOquery: a bridge between the Gene Expression Omnibus (GEO) and BioConductor. *Bioinformatics* 23 1846–1847. 10.1093/bioinformatics/btm254 17496320

[B8] DeSantis C. E. Ma J. Gaudet M. M. Newman L. A. Miller K. D. Goding Sauer A. (2019). Breast cancer statistics, 2019. *CA Cancer J. Clin.* 69 438–451. 31577379 10.3322/caac.21583

[B9] Du J. Yuan Z. Ma Z. Song J. Xie X. Chen Y. (2014). KEGG-PATH: Kyoto encyclopedia of genes and genomes-based pathway analysis using a path analysis model. *Mol. Biosyst.* 10 2441–2447. 10.1039/c4mb00287c 24994036

[B10] Early Breast, Cancer Trialists, Collaborative Group Davies C. Godwin J. Gray R. (2011). Relevance of breast cancer hormone receptors and other factors to the efficacy of adjuvant tamoxifen: patient-level meta-analysis of randomised trials. *Lancet* 378 771–784. 10.1016/s0140-6736(11)60993-8 21802721 PMC3163848

[B11] Fan M. Yan P. S. Hartman-Frey C. Chen L. Paik H. Oyer S. L. (2006). Diverse gene expression and DNA methylation profiles correlate with differential adaptation of breast cancer cells to the antiestrogens tamoxifen and fulvestrant. *Cancer Res.* 66 11954–11966. 10.1158/0008-5472.can-06-1666 17178894

[B12] Feng H. Gu Z. Y. Li Q. Liu Q. H. Yang X. Y. Zhang J. J. (2019). Identification of significant genes with poor prognosis in ovarian cancer via bioinformatical analysis. *J. Ovarian Res.* 12:35. 10.1186/s13048-019-0508-2 PMC6477749 31010415

[B13] Goodsell D. S. (2002). The molecular perspective: tamoxifen and the estrogen receptor. *Stem Cells* 20 267–268. 10.1634/stemcells.20-3-267 12004085

[B14] Guo Y. Zhang X. Yang M. Miao X. Shi Y. Yao J. (2010). Functional evaluation of missense variations in the human MAD1L1 and MAD2L1 genes and their impact on susceptibility to lung cancer. *J. Med. Genet.* 47 616–622. 10.1136/jmg.2009.074252 20516147

[B15] Gyorffy B. Lanczky A. Eklund A. C. Denkert C. Budczies J. Li Q. (2010). An online survival analysis tool to rapidly assess the effect of 22,277 genes on breast cancer prognosis using microarray data of 1,809 patients. *Breast Cancer Res. Treat.* 123 725–731. 10.1007/s10549-009-0674-9 20020197

[B16] Huang, da W. Sherman B. T. Lempicki R. A. (2009). Systematic and integrative analysis of large gene lists using DAVID bioinformatics resources. *Nat. Protoc.* 4 44–57. 10.1038/nprot.2008.211 19131956

[B17] Jelovac D. Macedo L. Goloubeva O. G. Handratta V. Brodie A. M. (2005). Additive antitumor effect of aromatase inhibitor letrozole and antiestrogen fulvestrant in a postmenopausal breast cancer model. *Cancer Res.* 65 5439–5444. 10.1158/0008-5472.can-04-2782 15958593

[B18] Jordan V. C. Brodie A. M. (2007). Development and evolution of therapies targeted to the estrogen receptor for the treatment and prevention of breast cancer. *Steroids* 72 7–25. 10.1016/j.steroids.2006.10.009 17169390 PMC2566956

[B19] Lai A. C. Crews C. M. (2017). Induced protein degradation: an emerging drug discovery paradigm. *Nat. Rev. Drug Discov.* 16 101–114. 10.1038/nrd.2016.211 27885283 PMC5684876

[B20] Lee J. H. Horak C. E. Khanna C. Meng Z. Yu L. R. Veenstra T. D. (2008). Alterations in Gemin5 expression contribute to alternative mRNA splicing patterns and tumor cell motility. *Cancer Res.* 68 639–644. 10.1158/0008-5472.can-07-2632 18245461 PMC2678556

[B21] Li X. P. Jiao J. U. Lu L. I. Zou Q. Zhu S. Zhang Y. (2016). Overexpression of ribosomal L1 domain containing 1 is associated with an aggressive phenotype and a poor prognosis in patients with prostate cancer. *Oncol. Lett.* 11 2839–2844. 10.3892/ol.2016.4294 27073561 PMC4812176

[B22] Magnuson T. Epstein C. J. (1984). Oligosyndactyly: a lethal mutation in the mouse that results in mitotic arrest very early in development. *Cell* 38 823–833. 10.1016/0092-8674(84)90277-0 6091901

[B23] Massenet S. Pellizzoni L. Paushkin S. Mattaj I. W. Dreyfuss G. (2002). The SMN complex is associated with snRNPs throughout their cytoplasmic assembly pathway. *Mol. Cell Biol.* 22 6533–6541. 10.1128/mcb.22.18.6533-6541.2002 12192051 PMC135628

[B24] Naorem L. D. Muthaiyan M. Venkatesan A. (2019). Integrated network analysis and machine learning approach for the identification of key genes of triple-negative breast cancer. *J. Cell. Biochem.* 120 6154–6167. 10.1002/jcb.27903 30302816

[B25] Payne E. M. Bolli N. Rhodes J. Abdel-Wahab O. I. Levine R. Hedvat C. V. (2011). Ddx18 is essential for cell-cycle progression in zebrafish hematopoietic cells and is mutated in human AML. *Blood* 118 903–915. 10.1182/blood-2010-11-318022 21653321 PMC3148170

[B26] Pineiro D. Fernandez-Chamorro J. Francisco-Velilla R. Martinez-Salas E. (2015). Gemin5: a multitasking RNA-binding protein involved in translation control. *Biomolecules* 5 528–544. 10.3390/biom5020528 25898402 PMC4496684

[B27] Pines J. (2011). Cubism and the cell cycle: the many faces of the APC/C. *Nat. Rev. Mol. Cell Biol.* 12 427–438. 10.1038/nrm3132 21633387

[B28] Rani A. Stebbing J. Giamas G. Murphy J. (2019). Endocrine resistance in hormone receptor positive breast cancer-from mechanism to therapy. *Front. Endocrinol.* 10:245. 10.3389/fendo.2019.00245 31178825 PMC6543000

[B29] Razandi M. Pedram A. Greene G. L. Levin E. R. (1999). Cell membrane and nuclear estrogen receptors (ERs) originate from a single transcript: studies of ERalpha and ERbeta expressed in Chinese hamster ovary cells. *Mol. Endocrinol.* 13 307–319. 10.1210/me.13.2.307 9973260

[B30] Redmond A. M. Byrne C. Bane F. T. Brown G. D. Tibbitts P. O’Brien K. (2015). Genomic interaction between ER and HMGB2 identifies DDX18 as a novel driver of endocrine resistance in breast cancer cells. *Oncogene* 34 3871–3880. 10.1038/onc.2014.323 25284587

[B31] Shannon P. Markiel A. Ozier O. Baliga N. S. Wang J. T. Ramage D. (2003). Cytoscape: a software environment for integrated models of biomolecular interaction networks. *Genome Res.* 13 2498–2504. 10.1101/gr.1239303 14597658 PMC403769

[B32] Szklarczyk D. Franceschini A. Wyder S. Forslund K. Heller D. Huerta-Cepas J. (2015). STRING v10: protein-protein interaction networks, integrated over the tree of life. *Nucleic Acids Res.* 43 D447–D452. 25352553 10.1093/nar/gku1003 PMC4383874

[B33] Vogelstein B. Papadopoulos N. Velculescu V. E. Zhou S. Diaz L. A. Jr. (2013). Cancer genome landscapes. *Science* 339 1546–1558. 10.1126/science.1235122 23539594 PMC3749880

[B34] Wang Z. Katsaros D. Shen Y. Fu Y. Canuto E. M. Benedetto C. (2015). Biological and clinical significance of MAD2L1 and BUB1, genes frequently appearing in expression signatures for breast cancer prognosis. *PLoS One* 10:e0136246. 10.1371/journal.pone.0136246 26287798 PMC4546117

[B35] Wild T. Budzowska M. Hellmuth S. Eibes S. Karemore G. Barisic M. (2018). Deletion of APC7 or APC16 allows proliferation of human cells without the spindle assembly checkpoint. *Cell Rep.* 25 2317.e8–2328.e5. 30485802 10.1016/j.celrep.2018.10.104 PMC6289045

[B36] Wirth K. G. Ricci R. Gimenez-Abian J. F. Taghybeeglu S. Kudo N. R. Jochum W. (2004). Loss of the anaphase-promoting complex in quiescent cells causes unscheduled hepatocyte proliferation. *Genes Dev.* 18 88–98. 10.1101/gad.285404 14724179 PMC314282

[B37] Yang F. Liu H. Zhao J. Ma X. Qi W. (2020). POLR1B is upregulated and promotes cell proliferation in non-small cell lung cancer. *Oncol. Lett.* 19 671–680. 31897183 10.3892/ol.2019.11136 PMC6924162

[B38] Yasar P. Ayaz G. User S. D. Gupur G. Muyan M. (2017). Molecular mechanism of estrogen-estrogen receptor signaling. *Reprod. Med. Biol.* 16 4–20. 10.1002/rmb2.12006 29259445 PMC5715874

[B39] Zhu X. F. Yi M. He J. Tang W. Lu M. Y. Li T. (2017). Pathological significance of MAD2L1 in breast cancer: an immunohistochemical study and meta analysis. *Int. J. Clin. Exp. Pathol.* 10 9190–9201. 31966791 PMC6965997

